# A diagnostic model for differentiating tuberculous spondylitis from pyogenic spondylitis: a retrospective case–control study

**DOI:** 10.1038/s41598-023-36965-w

**Published:** 2023-06-26

**Authors:** Yu Xi Liu, Fei Lei, Li Peng Zheng, Hao Yuan, Qing Zhong Zhou, Da Xiong Feng

**Affiliations:** Department of Orthopaedics, The Affiliated Hospital of Southwest Medical University, No. 25 Taiping Street, Lu Zhou City, China

**Keywords:** Neuroscience, Diseases, Medical research

## Abstract

The purpose of this study was to describe and compare the clinical data, laboratory examination and imaging examination of tuberculous spondylitis (TS) and pyogenic spondylitis (PS), and to provide ideas for diagnosis and treatment intervention. The patients with TS or PS diagnosed by pathology who first occurred in our hospital from September 2018 to November 2021 were studied retrospectively. The clinical data, laboratory results and imaging findings of the two groups were analyzed and compared. The diagnostic model was constructed by binary logistic regression. In addition, an external validation group was used to verify the effectiveness of the diagnostic model. A total of 112 patients were included, including 65 cases of TS with an average age of 49 ± 15 years, 47 cases of PS with an average of 56 ± 10 years. The PS group had a significantly older age than the TS group (P = 0.005). In laboratory examination, there were significant differences in WBC, neutrophil (N), lymphocyte (L), ESR, CRP, fibrinogen (FIB), serum albumin (A) and sodium (Na). The difference was also statistically significant in the comparison of imaging examinations at epidural abscesses, paravertebral abscesses, spinal cord compression, involvement of cervical, lumbar and thoracic vertebrae. This study constructed a diagnostic model, which was Y (value of TS > 0.5, value of PS < 0.5) = 1.251 * X1 (thoracic vertebrae involved = 1, thoracic vertebrae uninvolved = 0) + 2.021 * X2 (paravertebral abscesses = 1, no paravertebral abscess = 0) + 2.432 * X3 (spinal cord compression = 1, no spinal cord compression = 0) + 0.18 * X4 (value of serum A)−4.209 * X5 (cervical vertebrae involved = 1, cervical vertebrae uninvolved = 0)−0.02 * X6 (value of ESR)−0.806 * X7 (value of FIB)−3.36. Furthermore, the diagnostic model was validated using an external validation group, indicating a certain value in diagnosing TS and PS. This study puts forward a diagnostic model for the diagnosis of TS and PS in spinal infection for the first time, which has potential guiding value in the diagnosis of them and provides a certain reference for clinical work.

## Introduction

Spinal infection may involve the vertebrae, the intervertebral discs, and the adjacent intraspinal and paraspinal soft tissues^1^. During the development of the disease, the formation of abscesses or edemas can destroy vertebrae or cause neurologic disorders. Recently, the incidence of spondylitis has increased due to longer life expectancy of patients with chronic debilitating diseases^2–4^.

Tuberculous spondylitis (TS) results from dissemination of the tuberculous bacilli from a distant active source or as a result of latent reactivation, and pyogenic spondylodiscitis (PS) refers to infectious spondylodiscitis caused by ordinary bacteria. Differentiation between TS and PS is essential for deciding on the appropriate therapeutic regimen. Although bacterial culture is considered the gold standard for diagnosing infectious diseases, it may have low positivity rates due to unreasonable antibiotic use before tissue sampling or difficulty in culturing the causative agent^5,6^. Meanwhile, spinal infection symptoms often have a non-specific and insidious onset, and identifying the etiological agent can be elusive in about a third of spinal infection cases^7^. Although various diagnostic techniques such as computed tomography (CT), magnetic resonance imaging (MRI) and positron emission tomography have been used in clinical practice, there are still limitations^8–10^. CT has a good sensitivity in evaluating bony changes, but it may not be sufficient for some patients with TS who lack typical sequestra and pathological calcifications^11^. In contrast, MRI with gadolinium enhancement is widely recognized as the most reliable method for diagnosing spinal infection. However, while there are features that can suggest either TS or PS, there is a significant overlap between the two. As for positron emission tomography CT with F‑18 fluorodeoxyglucose (^18^FDG PET‑CT), it can only suggest an infectious disease and has limited value in differentiating between TS and PS^12,13^.

The aim of this study was to describe and compare the clinical data, laboratory examination and imaging examination of TS and PS, and to provide a diagnostic model for diagnosis and treatment intervention.

## Materials and methods

**Figure 1:**
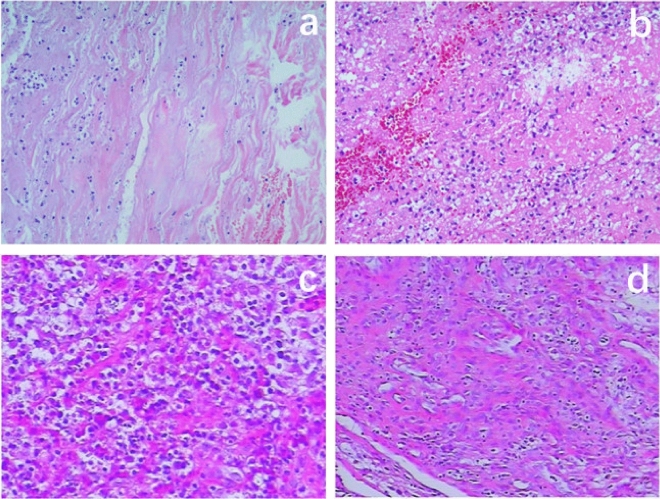
Pathological specimens of tuberculous spondylitis and pyogenic spondylodiscitis. (**a**) and (**b**) Pathological specimens of tuberculous spondylitis. Visible granulomatous inflammation and caseous necrosis. (**c**) and (**d**) Pathological specimens of pyogenic spondylodiscitis. Visible chronic suppurative inflammation with extensive proliferation of inflammatory granulation tissue.

The current study conformed to the principles drafted in the Helsinki declaration and was approved by the medical ethical committee of the Affiliated Hospital of Southwest Medical University (approval number: KY2022262). The need for consent was waived by the ethical committee of the Affiliated Hospital of Southwest Medical University due to the retrospective nature of this study. The study evaluated the clinical, laboratory and imaging data of 65 patients with TS (the TS group) and 47 patients with PS (the PS group) in a single institution from September 2018 to November 2021. And 25 TS patients (the EV-TS group) and 21 PS patients (the EV-PS group) who had continuous medical visits from December 2021 to April 2023 were used as an external validation group to verify the diagnostic model. All patients diagnosed by pathology (Fig. 1). The inclusion criteria included the following: age ≥ 18 years old; admitted to hospital for the first time because of spinal infection; cervical, thoracic or lumbosacral vertebrae infection. The exclusion criteria included the following: infection caused by invasive examination or surgery of the spine; previous surgical history of spinal infection; complication with tumor or autoimmune disease; complication with bone or joint infection in other parts; and incomplete medical record or radiological examination.

Clinical information including sex, age and course of disease were retrieved from the electronic medical record system for patients. Laboratory examination: fasting venous blood was collected at 6:00 a.m. the day after admission to collect blood infection markers, function of blood coagulation, kidney function tests, liver function tests and serum electrolytes. The blood infection markers included leukocyte (WBC), neutrophil (N), eosinophil (E), basophil (B), lymphocyte (L), monocyte (M), erythrocyte sedimentation rate (ESR) and C-reactive protein (CRP). The function of blood coagulation included platelet (PLT), prothrombin time (PT), activated partial thromboplastin time (APTT), prothrombin time (TT) and fibrinogen (FIB). The kidney function tests included creatinine (Cr) and glomerular filtration rate (GFR). The liver function tests included albumin (A), globulin (G), alanine transaminase (ALT) and aspartate transaminase (AST). The serum electrolytes included potassium (K), sodium (Na), calcium (Ca) and chlorine (Cl).

Imaging assessment on admission included computed tomography scans (CT) and magnetic resonance imaging (MRI). The following lesions were sought on CT images: (1) disc space narrowing; (2) bone destruction of vertebral body; (3) destruction of pedicle bone. The following lesions were sought on MRI: (1) vertebral involvement (single-segment, multiple-segments, cervical, thoracic, lumbar, or sacral); (2) location and quantity of abscesses (unifocal, multifocal, discal, epidural, or paravertebral); (3) spinal cord compression, radicular compression; (4) cord signal changes (changing on both T1- and T2-weighted images). All the images were reviewed by a senior radiologist specialized in musculoskeletal imaging.

## Statistical analyses

Statistical analyses were performed using the Statistical Package for the Social Sciences software version 23.0 (IBM Corp., Armonk, NY). Continuous data were expressed as mean ± standard deviation or median and interquartile range [M (P_25_, P_75_)], while categorical data were expressed as the number of patients (percentage). Continuous data were compared between the two groups using the independent t-test or Mann–Whitney U test, while categorical data were compared using the Pearson χ^2^ test or Fisher’s exact test. Multi-factor analysis used binary logistic regression and screened independent variables by stepwise method. All statistical analyses were two-tailed and the significance level was set at P value < 0.05.

## Results

The clinical and laboratory data of the TS and PS groups were depicted in Table 1. Clinically, there was no significant difference in gender and course of disease between the TS and PS groups (p > 0.05). The PS group had a significantly older age than the TS group (56 ± 10 years vs. 49 ± 15 years, P = 0.005). Based on the laboratory results, the PS group had a significantly greater WBC counts (6550 ± 1960/mm^3^ vs. 8690 ± 3430/mm^3^, P < 0.000), N counts (4710 ± 1750/mm^3^ vs. 6590 ± 3190/mm^3^, P < 0.000), L counts (1160 ± 500/mm^3^ vs. 1420 ± 6100/mm^3^, P = 0.014), ESR level (20 ± 19 mm/h vs. 82 ± 32 mm/h, P < 0.000) and CRP level (19.87 ± 19.00 mg/L vs. 45.46 ± 48.73 mg/L, P = 0.001). The FIB level of the function of blood coagulation was also significantly greater in the PS group than in the TS group (6.12 ± 1.71 g/L vs. 4.67 ± 1.27 g/L, P < 0.001). However, the serum A level (4.0 ± 0.4 g/dL vs. 3.8 ± 0.4 g/dL, P = 0.005) of the liver function tests and the serum Na level (141.6 ± 3.1 mmol/L vs. 139.8 ± 3.3 mmol/L, P = 0.003) of the serum electrolytes were significantly greater in the TS group than in the PS group.

**Table 1:** Clinical data and laboratory examination of the tuberculous spondylitis group and pyogenic spondylodiscitis group.

	Tuberculous group (n = 65)	Pyogenic group (n = 47)	t/χ^2^/Z Value	p Value
Age (years)	49 ± 15	56 ± 10	− 2.874	0.005
Men/Women	37/28	30/17	0.541	0.462
Course of disease (months)	6.0 (2.5, 12.0)	4.0 (2.0, 12.0)	− 1.604	0.109
Blood infection markers				
WBC (/mm^3^)	6550 ± 1960	8690 ± 3430	− 3.857	< 0.000
N (/mm^3^)	4710 ± 1750	6590 ± 3190	− 3.667	< 0.000
E (/mm^3^)	140 ± 100	120 ± 120	0.916	0.362
B (/mm^3^)	30 ± 30	30 ± 10	0.814	0.417
L (/mm^3^)	1160 ± 500	1420 ± 610	− 2.487	0.014
M (/mm^3^)	520 ± 190	530 ± 240	− 0.343	0.732
ESR (mm/h)	20 ± 19	82 ± 32	− 11.699	< 0.000
CRP (mg/L)	19.87 ± 19.00	45.46 ± 48.73	− 3.417	0.001
Function of blood coagulation				
PLT (/mm^3^)	256,000 ± 64,000	290,000 ± 106,000	− 1.977	0.052
PT (s)	12.2 ± 1.3	12.5 ± 1.4	− 1.088	0.279
APTT (s)	30.2 ± 5.5	30.4 ± 5.7	− 0.144	0.886
TT (s)	16.9 ± 0.9	17.0 ± 1.1	− 0.525	0.600
FIB (g/L)	4.67 ± 1.27	6.12 ± 1.71	− 4.931	< 0.000
Kidney function tests				
Cr (μmol/L)	61.9 ± 16.2	59.1 ± 16.1	0.918	0.361
GFR (mL/min)	107.3 ± 19.2	105.9 ± 13.5	0.456	0.649
Liver function tests				
A (g/dL)	4.0 ± 0.4	3.7 ± 0.4	2.871	0.005
G (g/dL)	3.0 ± 0.4	3.1 ± 0.7	− 0.615	0.541
ALT (U/L)	28.1 ± 39.6	38.0 ± 36.9	− 1.351	0.179
AST (U/L)	29.0 ± 29.8	28.5 ± 21.0	0.095	0.925
Serum electrolytes				
K (mmol/L)	4.22 ± 0.53	4.17 ± 0.41	0.566	0.572
Na (mmol/L)	141.6 ± 3.1	139.8 ± 3.3	2.987	0.003
Ca (mmol/L)	2.25 ± 0.13	2.22 ± 0.12	1.139	0.257
Cl (mmol/L)	106.3 ± 3.5	105.1 ± 3.9	1.841	0.068

WBC indicates leukocyte; N, neutrophil; E, eosinophil; B, basophil; L, lymphocyte; M, monocyte; ESR, erythrocyte sedimentation rate; CRP, C-reactive protein; PLT, platelet; PT, prothrombin time; APTT, activated partial thromboplastin time; TT, prothrombin time; FIB, fibrinogen; Cr, creatinine; GFR, glomerular filtration rate; A, albumin; G, globulin; ALT, alanine transaminase; AST, aspartate transaminase; K, potassium; Na, potassium; Ca, calcium; Cl, chlorine. p-values derived from independent t-test, Pearson χ^2^ test and Mann–Whitney U test.

The radiological characteristics of the TS and PS groups were summarized in Table 2. In the PS group, the proportions of cervical (n = 6 [12.8%] vs. n = 1 [1.5%], P = 0.043) and lumbar (n = 35 [74.5%] vs. n = 34 [52.3%], P = 0.017) vertebrae involved were higher than those in the TS group, while the proportions of thoracic (n = 13 [27.7%] vs. n = 41 [63.1%], P < 0.000) vertebrae involved, epidural abscesses (n = 6 [12.8%] vs. n = 23 [35.4%], P = 0.007), paravertebral abscesses (n = 18 [38.3%] vs. n = 49 [75.4%], P < 0.000) and spinal cord compression (n = 9 [19.1%] vs. n = 37 [56.9%], P < 0.000) were lower than those in the TS group.

**Table 2:** Imaging examination of the tuberculous spondylitis group and pyogenic spondylodiscitis group.

	Tuberculous group (n = 65)	Pyogenic group (n = 47)	t/χ^2^ Value	p Value
CT				
Disc space narrowing	54 (83.1)	33 (70.2)	2.603	0.107
Bone destruction of vertebral body	65 (100.0)	47 (100.0)	–	–
Destruction of pedicle bone	11 (16.9)	3 (6.4)	2.771	0.096
MRI				
Vertebral involvement				
Single/Multiple-segment	3/62	2/45	0.000	1.000
Cervical	1 (1.5)	6 (12.8)	4.108	0.043
Thoracic	41 (63.1)	13 (27.7)	13.704	< 0.000
Lumbar	34 (52.3)	35 (74.5)	5.663	0.017
Sacral	7 (10.8)	7 (10.8)	0.424	0.515
Abscesses				
Unifocal	4 (6.2)	6 (12.8)	0.766	0.381
Multifocal	52 (80.0)	25 (53.2)	9.125	0.003
Discal	35 (53.8)	25 (53.2)	0.005	0.945
Epidural	23 (35.4)	6 (12.8)	7.273	0.007
Paravertebral	49 (75.4)	18 (38.3)	15.609	< 0.000
Spinal cord compression	37 (56.9)	9 (19.1)	16.081	< 0.000
Radicular compression	9 (13.8)	3 (6.4)	1.588	0.208
Cord signal changes	4 (6.2)	2 (4.3)	0.000	0.988

p-values derived from Pearson χ^2^ test and Fisher exact test.

**Figure 2:**
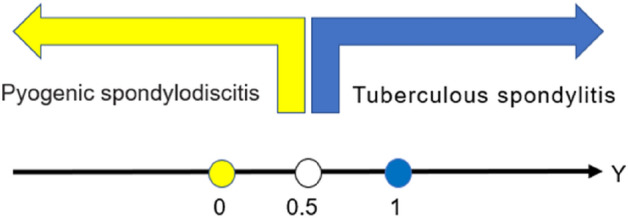
Diagnostic result corresponding to Y value.

The result of multivariate analysis by binary logistic regression were depicted in Table 3, and screened independent variables by stepwise method. Using this regression model proposed a diagnostic model that Y (value of TS > 0.5, value of PS < 0.5) (Fig. 2) = 1.251 * X1 (thoracic vertebrae involved = 1, thoracic vertebrae uninvolved = 0) + 2.021 * X2 (paravertebral abscesses = 1, no paravertebral abscess = 0) + 2.432 * X3 (spinal cord compression = 1, no spinal cord compression = 0) + 0.18 * X4 (value of serum A)−4.209 * X5 (cervical vertebrae involved = 1, cervical vertebrae uninvolved = 0)−0.02 * X6 (value of ESR)−0.806 * X7 (value of FIB)−3.36.

**Table 3:** Multivariate analysis of clinical, laboratory and radiological factors affecting diagnosis.

Variables	Odds ratio	95% confidence interval		p Value
ESR	0.980	0.958	1.003	0.082
FIB	0.446	0.276	0.723	0.001
A	1.197	1.014	1.413	0.034
Vertebral involvement				
Cervical	0.015	0.000	0.559	0.023
Thoracic	3.495	0.994	12.291	0.051
Paravertebral abscesses	7.543	1.962	29.003	0.003
Spinal cord compression	11.382	2.494	51.958	0.002

ESR indicates erythrocyte sedimentation rate; FIB, fibrinogen; A, albumin.

p-values derived from binary logistic regression.

The diagnostic model was tested with the original data, and the diagnostic value for TS and PS was presented in Table 4. Additionally, statistical measures of the diagnostic model's performance in diagnosing TS and PS were compared in Table 5. For the diagnosis of TS, the diagnostic model demonstrated 0.85 sensitivity, 0.85 specificity, 0.89 positive predictive value, 0.80 negative predictive value, and 0.85 accuracy. In the diagnosis of PS, the sensitivity, specificity, positive predictive value, negative predictive value, and accuracy were 0.85, 0.85, 0.80, 0.89, and 0.85, respectively. Furthermore, the model’s performance was validated externally using a separate dataset, and the results were presented in Table 6. The diagnostic model’s performance in diagnosing TS and PS in the external validation group was also described in Table 7. The diagnostic model achieved 0.84 sensitivity, 0.86 specificity, 0.88 positive predictive value, 0.82 negative predictive value, and 0.85 accuracy in diagnosing EV-TS, and 0.86 sensitivity, 0.84 specificity, 0.82 positive predictive value, 0.88 negative predictive value, and 0.85 accuracy in diagnosing EV-PS. These results suggest that the diagnostic model has significant potential in the diagnosis of both TS and PS.

**Table 4:** The value of diagnostic model in the diagnosis of tuberculous spondylitis and pyogenic spondylodiscitis.

Diagnostic model	Pathology		Total
	TS	PS	
TS	55	7	62
PS	10	40	50
Total	65	47	112

TS indicates tuberculous spondylitis; PS, pyogenic spondylodiscitis.

**Table 5:** Comparison of statistical indexes of the diagnostic model in the diagnosis of tuberculous spondylitis and pyogenic spondylodiscitis.

Disease	Se	Sp	PPV	NPV	Ac
TS	0.85	0.85	0.89	0.80	0.85
PS	0.85	0.85	0.80	0.89	0.85

TS indicates tuberculous spondylitis; PS, pyogenic spondylodiscitis; Se, sensiti-vity; Sp, specificity; PPV, positive predictive value; NPV, negative predictive value; Ac, accu-racy.

**Table 6:** External validation of the value of diagnostic model in the diagnosis of tuberculous spondylitis and pyogenic spondylodiscitis.

Diagnostic model	Pathology		Total
	TS	PS	
TS	21	3	24
PS	4	18	22
Total	25	21	46

TS indicates tuberculous spondylitis; PS, pyogenic spondylodiscitis.

**Table 7:** The diagnostic performance of the diagnostic model in the external validation group of tuberculous spondylitis and pyogenic spondylodiscitis.

Disease	Se	Sp	PPV	NPV	Ac
TS	0.84	0.86	0.88	0.82	0.85
PS	0.86	0.84	0.82	0.88	0.85

TS indicates tuberculous spondylitis; PS, pyogenic spondylodiscitis; Se, sensiti-vity; Sp, specificity; PPV, positive predictive value; NPV, negative predictive value; Ac, accu-racy.

## Discussion

Although there have been several previous studies^8–10^ describing the characteristics of TS and PS, our comparative analysis of the two yields fifteen meaningful differentiating variables. However, no single diagnostic model is specific to either disease. Our diagnostic model is therefore a useful diagnostic tool that can help differentiate between TS and PS. A literature search reveals that our diagnostic model is the first of its kind for the differential diagnosis of TS and PS.

For laboratory findings, known risk factors for PS include greater levels of WBC counts, neutrophil counts, ESR and CRP^8^. Our study also found that L counts and FIB level might be valuable laboratory findings to diagnose spondylodiscitis. Elevated FIB levels often coincide with elevated acute-phase proteins, such as in bacterial infections like pneumonia, rheumatic fever, sepsis, etc. FIB interacts with inflammatory factors to affect the occurrence and development of diseases^14,15^. Univariate analyses indicated that greater levels of these biomarkers were suggestive of pyogenic rather than tuberculous spondylodiscitis. Meanwhile, greater levels of serum A and Na were suggestive of tuberculous rather than pyogenic spondylodiscitis. However, the main function of serum Na is to maintain extracellular fluid volume, osmotic pressure and acid–base balance, and it also plays a role in maintaining normal muscle and nerve excitability.

**Figure 3:**
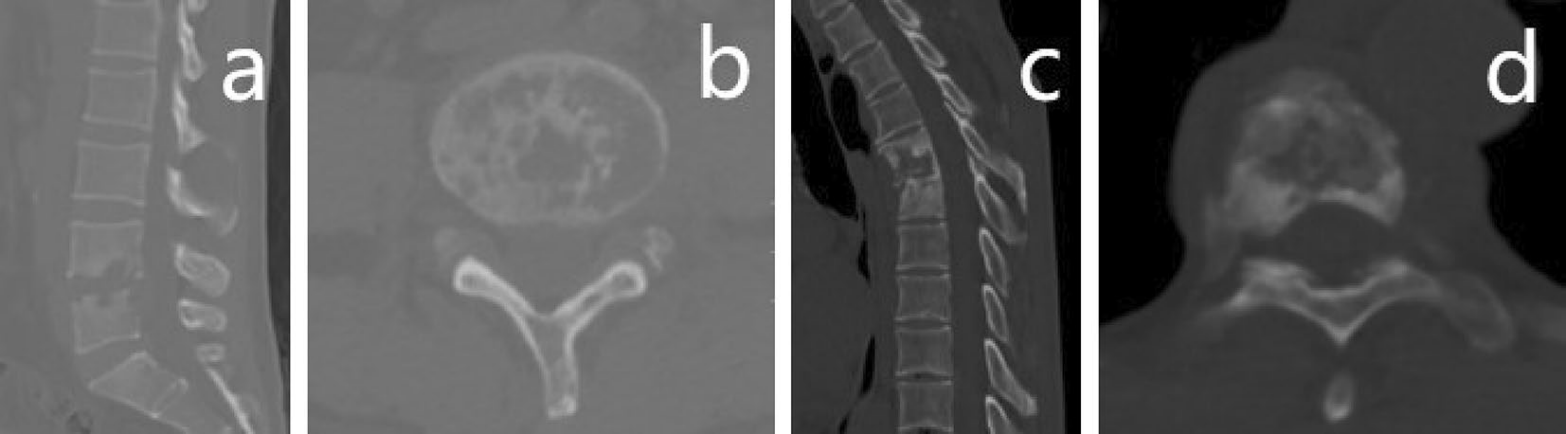
CT scan images of tuberculous spondylitis and pyogenic spondylitis. (**a** and **b**) CT scan images of pyogenic spondylitis. Lumbar spine involvement is more common in pyogenic spondylitis. (**c** and **d**) CT scan images of tuberculous spondylitis. Thoracic spine involvement is more common in tuberculous spondylitis.

Hematogenous spread accounts for more than 50% of spinal infections^16^. In most cases of TS and PS, pathogenic microorganisms remain near vertebral endplates, and then spread through the intervertebral discs to adjacent vertebral bodies and accessory structures. There is no blood supply in adult intervertebral discs^17^, and nutrition is contingent on diffusion from the endplates. When the endplates are damaged, the intervertebral space will narrow or possible obliterate. Our study found no significant differences in the intervertebral disc spaces between TS and PS, but Galhotra^18^ did. Lumbar segments are likely to be involved in PS, which could be due to their hematogenous spread, reflecting, to some extent, the vascular supply to lumbar structures^19^. This localization may also be related to the high mobility and load forces in the lumbar region^20^. In our study, 74.5% of PS patients had lumbar spine involvement (Fig. 3a and b) compared to 52.3% of TS patients and the difference was statistically significant. Thoracolumbar levels are commonly involved in TS, which may be explained by the coexistence of pulmonary tuberculosis as the origin of the hematogenous spread. In our study, 63.1% of TS patients had thoracic spine involvement (Fig. 3c and d), while this figure was only 27.7% in PS patients, and the difference was statistically significant.

**Figure 4:**
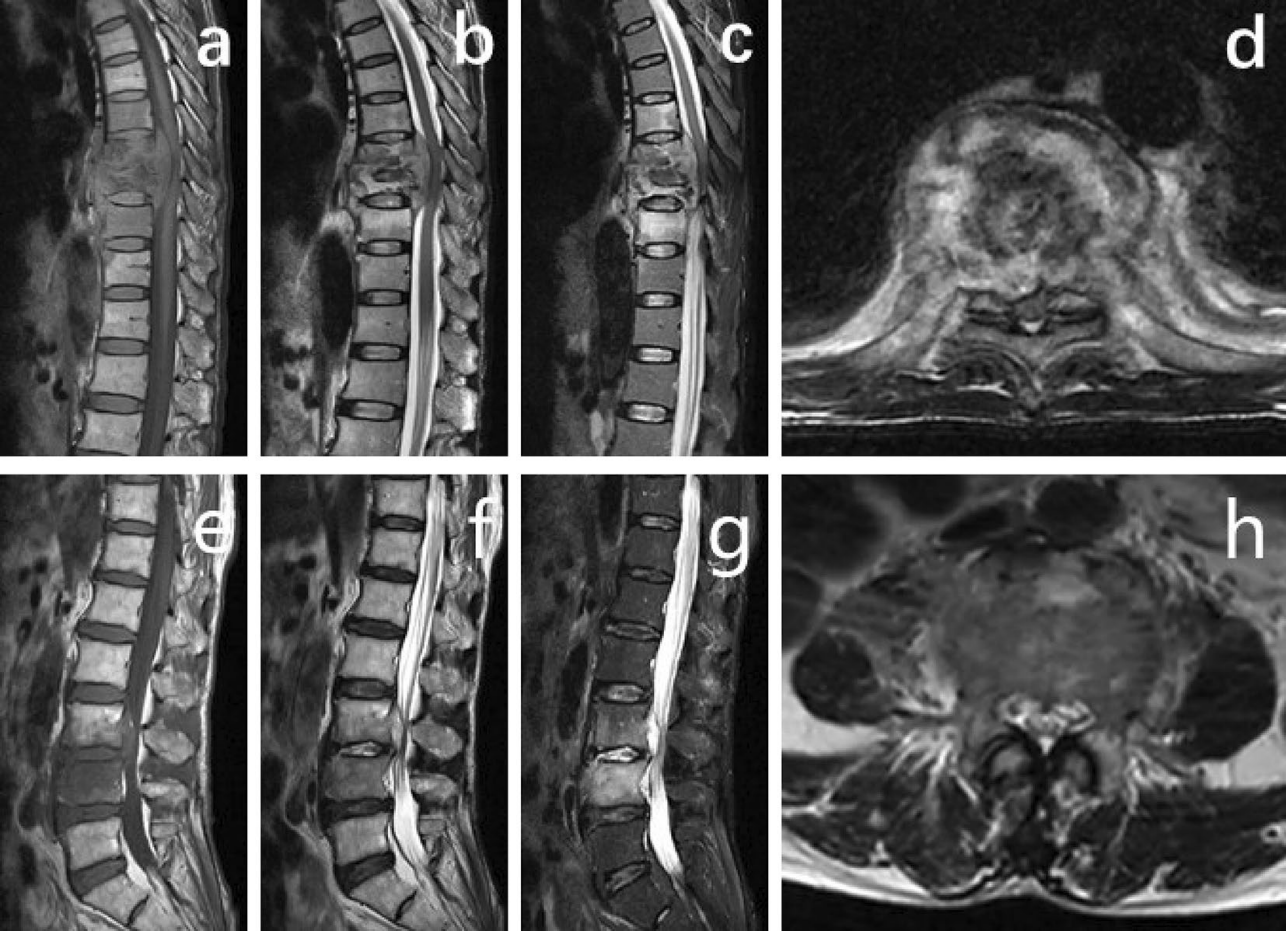
MRI scan images of tuberculous spondylitis and pyogenic spondylitis. (**a**–**d**) MRI scan images of tuberculous spondylitis. (**e**–**h**) MRI scan images of pyogenic spondylitis. In the abscesses, tuberculous spondylitis is more likely than pyogenic spondylitis to involve multiple segments with paravertebral spread.

In the abscesses, TS is more likely than PS to involve multiple segments with paravertebral spread and this is in agreement with our findings (Fig. 4). Previous studies^21^ revealed that TS had a higher incidence rate of epidural abscess formation than PS. In our study, 35.4% of TS patients had epidural abscess formation compared with 12.8% of PS patients, while 37 TS patients (56.9%) and 9 PS patients (19.1%) presented with imaging signs of spinal cord compression, and both differences were statistically significant.

The above mentioned clinical characteristics are statistically significant and clinically correlated with the type of infection and can be used as efficient indicators for the differential diagnosis^22,23^. Logistic regression analysis is usually used as the model for differential diagnosis. In this study, we included three continuous variables and four categorical variables in the diagnostic model that yields great total accuracy for TS and PS diagnosis. However, there are limitations to our study. It is a retrospective study, with cases limited to a single Chinese institution. Moreover, it is limited to inpatients with relatively advanced disease. Our diagnostic model is less useful early in the disease process, where many of the findings have not yet developed. In addition, the primary aim of this study was to construct a more widely applicable diagnostic model for clinical diagnosis and differential diagnosis of TS and PS by identifying differences in common laboratory and imaging examinations between the two. However, there were limitations in exploring their unknown differences.

## Conclusion

This study puts forward a diagnostic model for the diagnosis of TS and PS in spinal infection for the first time, which has potential guiding value in the diagnosis of them and provides a certain reference for clinical work.

## Supplementary Information

Supplementary Information 1.

Supplementary Information 2.

Supplementary Information 3.

Supplementary Information 4.

## Data Availability

All data generated or analysed during this study are included in this published article [and its supplementary information files].
